# Implementation of depression screening in antenatal clinics through tablet computers: results of a feasibility study

**DOI:** 10.1186/s12911-017-0459-8

**Published:** 2017-05-10

**Authors:** José S. Marcano-Belisario, Ajay K. Gupta, John O’Donoghue, Paul Ramchandani, Cecily Morrison, Josip Car

**Affiliations:** 1 Global eHealth Unit, Department of Primary Care and Public Health, School of Public Health, Imperial College London, London, UK; 2 Faculty of Medicine, National Heart and Lung Institute, Imperial College London, London, UK; 3 Global eHealth Unit, Department of Primary Care and Public Health, School of Public Health, Imperial College London, London, UK; 4 The Centre for Pyschiatry, Department of Medicine, Imperial College London, London, UK; 5 Global eHealth Unit, Department of Primary Care and Public Health, School of Public Health, Imperial College London, London, UK; 6 Global eHealth Unit, Department of Primary Care and Public Health, School of Public Health, Imperial College London, London, UK

**Keywords:** Antenatal depression, Mental health, Edinburgh Postnatal Depression Scale (EPDS), Whooley questions, Population screening, Patient self-report, Mobile health (mHealth), Apple ® iPad®, Tablet computers, Survey layout

## Abstract

**Background:**

Mobile devices may facilitate depression screening in the waiting area of antenatal clinics. This can present implementation challenges, of which we focused on survey layout and technology deployment.

**Methods:**

We assessed the feasibility of using tablet computers to administer a socio-demographic survey, the Whooley questions and the Edinburgh Postnatal Depression Scale (EPDS) to 530 pregnant women attending National Health Service (NHS) antenatal clinics across England. We randomised participants to one of two layout versions of these surveys: (i) a scrolling layout where each survey was presented on a single screen; or (ii) a paging layout where only one question appeared on the screen at any given time.

**Results:**

Overall, 85.10% of eligible pregnant women agreed to take part. Of these, 90.95% completed the study procedures. Approximately 23% of participants answered *Yes* to at least one Whooley question, and approximately 13% of them scored 10 points of more on the EPDS. We observed no association between survey layout and the responses given to the Whooley questions, the median EPDS scores, the number of participants at increased risk of self-harm, and the number of participants asking for technical assistance. However, we observed a difference in the number of participants at each EPDS scoring interval (*p* = 0.008), which provide an indication of a woman’s risk of depression. A scrolling layout resulted in faster completion times (median = 4 min 46 s) than a paging layout (median = 5 min 33 s) (*p* = 0.024). However, the clinical significance of this difference (47.5 s) is yet to be determined.

**Conclusions:**

Tablet computers can be used for depression screening in the waiting area of antenatal clinics. This requires the careful consideration of clinical workflows, and technology-related issues such as connectivity and security. An association between survey layout and EPDS scoring intervals needs to be explored further to determine if it corresponds to a survey layout effect. Future research needs to evaluate the effect of this type of antenatal depression screening on clinical outcomes and clinic workflows.

**Trial registration:**

This study was registered in ClinicalTrials.gov under the identifier NCT02516982 on 20 July 2015.

**Electronic supplementary material:**

The online version of this article (doi:10.1186/s12911-017-0459-8) contains supplementary material, which is available to authorized users.

## Background

The role of mobile technology for the assessment and screening of mental health disorders has received much attention in recent years [1–5]. Devices such as smartphones and tablet computers may facilitate the practical implementation of mental health population screening programmes in clinical settings. For patients, these devices could make the completion of screening scales more convenient, while increasing the perceived sense of confidentiality. The potential advantage for clinicians includes the rapid and convenient access to patients’ results. As recent evidence suggests that using mobile devices to administer validated screening scales does not affect their overall data equivalence (when compared to traditional delivery modes such as paper) [6], it is timely to explore the implementation of mobile-based screening.

Mobile-based screening programmes could be particularly beneficial for maternal mental health, especially antenatal depression. This condition is one of the most common during pregnancy, affecting between 7% and 12% of pregnant women [7, 8]. If left untreated, antenatal depression can have severe and long-lasting consequences for mothers, and their children and families [9–15]. Moreover, the antenatal period offers an opportunity of frequent interactions between pregnant women and their healthcare providers. Screening scales may be administered during this cycle of encounters as recommended by clinical guidelines [16].

Research has shown that the use of screening scales in the waiting area of clinical facilities can work well [17]. Patients who complete surveys whilst waiting for their appointment tend to report reduced frustration levels. Gathering data beforehand could also trigger meaningful discussions during the consultation that patients may have trouble raising directly [17]. Lastly, this could facilitate the monitoring of disease progression and treatment response, and help to ensure that important issues are not overlooked by the clinical team [17].

However, the use of mobile technology for screening in clinical settings presents implementation challenges of which we focus on two: survey design and deployment. Survey layout can influence respondents’ behaviour, a phenomenon known as survey layout effect [18–20]. *Scrolling layouts* (i.e., presenting all questions on a single screen) are thought to result in higher subjective ratings, lower breakoff and item non-response rates, fewer technical problems, and faster completion times [18] than *paging layouts* (i.e., presenting one question per screen). Although these findings relate to volunteer activities surveys, they could have clinical implications.

Depending on its size and direction, a survey layout effect on the subjective ratings given to clinical scales could result in changes to overall clinical assessments for reasons other than the construct under assessment. This could result in patients needing to undergo unnecessary diagnostic pathways due to false positive results, or in diagnostic delays due to false negative results. For clinical teams, there could be implications in terms of increased workload due to false positive results, or liability due to false negatives.

The findings from the survey methodology literature [18] also raise practical implications for the deployment of mobile technology in clinical settings. The potential for added workload for clinical staff, training and capacity needs, location and privacy of screening, network issues, and responsibility for technology are some of the perceived barriers to the success of mobile-based antenatal depression screening [5, 21]. Indicators from survey methodology studies (e.g., completion times, breakoff rates, technical problems) [18] may allow practitioners to capture useful information about the real impact of these barriers, giving opportunity to address them.

The aim of this study was to assess the feasibility of using tablet computers (Apple® iPads®) in the waiting area of antenatal clinics for implementing the recommendations of the National Institute for Health and Care Excellence (NICE) guidelines for recognising antenatal depression [16]. Our main outcomes were participants’ responses to the Whooley questions and the EPDS, and whether these differed from prevalence depression rates reported in the literature. In addition, we chose completion time and the number of participants requesting technical assistance as indicators of the impact that the deployment of mobile technology could have on the workload and schedules of clinics. Furthermore, we explored if there were any differences between two survey layouts across all study outcomes. We also assessed smartphone and tablet computer ownership amongst our participants. The latter can provide an indication of participants’ familiarity with mobile technology, and of the potential reach of future mobile health interventions. Breakoff rates were interpreted in relation to the incompatibility between research procedures and clinic schedules. Lastly, we report the deployment challenges that we identified during this feasibility study. We did not assess item non-response rates, as validation procedures did not allow participants to leave questions unanswered.

## Methods

For the protocol of this feasibility study see Marcano-Belisario and colleagues [22].

We assessed the feasibility of using tablet computers to administer a socio-demographic survey, the Whooley questions and the Edinburgh Postnatal Depression Scale (EPDS) to 530 pregnant women attending antenatal clinics in National Health Service (NHS) facilities across England. We used a randomised controlled study design to allocate participants to one of two layout versions of these surveys: a paging layout or a scrolling layout.

### Participants and participant recruitment

We recruited adult (18 years or older) pregnant women of any gestational age, pregnancy history and parity. We excluded women who had been diagnosed with depression or generalised anxiety disorder in the past 12 months (from the moment they were approached by the research team), or who were currently receiving treatment for any of these disorders. We also excluded women who did not feel comfortable reading and writing in English.

Recruitment took place between October 2015 and May 2016 using an opportunistic approach. Pregnant women attending antenatal clinics in any of the participating centres (i.e., antenatal clinics in general practices, community services and hospitals) were approached in the waiting area by a research midwife or a clinical studies officer (CSO) with information about the study and a copy of the participant information leaflet (PIL). Those consenting to take part in the study were then asked to complete the study procedures in the clinic’s waiting area before their appointment.

**Fig. 1:**
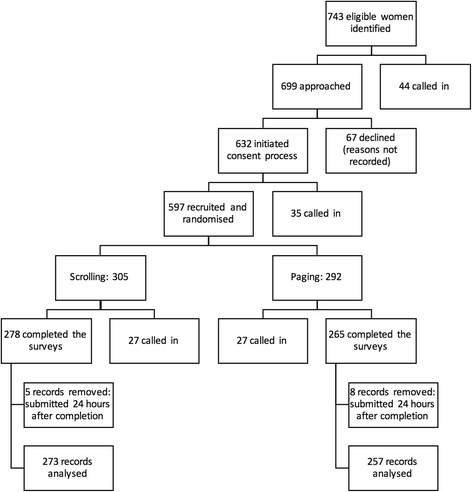
Study flow

As shown in Fig. 1, 699 eligible women were approached in the waiting area of participating clinics. Of these, 632 agreed to take part in the study and initiated the consent process. In relation to the total number of eligible participants (*N* = 743), our recruitment rate was 85.10%. However, 44 eligible women were called in before they could be approached by a research midwife or CSO. Therefore, our recruitment rate in relation to the total number of eligible women who were approached (*N* = 699) was 90.41%.

Of the 632 women who initiated the consent process, 35 were called in before they could complete the informed consent form. Therefore, 597 participants were recruited into the study. Of these, 54 were called in (27 participants from the *Scrolling* group and 27 participants from the *Paging* group) before completing the surveys. Therefore, our completion rate was 90.95% (543 participants out of the 597 who were initially randomised).

### Technology and materials

We designed the surveys in Snap® survey software [23], and administered them through Snap® Mobile app (versions 4.0.30 to 4.0.32) for Apple® iPhone Operating System (iOS^TM^) [24] running on Apple® iPad Air® and Apple® iPad mini^TM^ tablet computers. Responses were stored in Snap® WebHost [25]. These solutions supported the required survey layouts and offline data collection. The latter being important due to the variability in internet access across NHS facilities. We originally intended to use Apple® iPad Air® tablet computers to control for screen size; due to limited resources however, we had to use Apple® iPad mini^TM^ tablet computers in some sites.

### Surveys

We developed an 11-question socio-demographic survey to assess participants’ age group, ethnicity, relationship status, employment status, level of education, smartphone and tablet ownership, pregnancy history and parity, pregnancy trimester, and previous history of depression (Additional file 1).

Following NICE recommendations [16], we used the Whooley questions and the EPDS. The former is a 2-item survey evaluating the presence of depressed mood and anhedonia over the past month [26] (Additional file 2). An affirmative answer to either question should be followed up by further assessment using a validated screening tool such as the EPDS [16].

The EPDS is a 10-item instrument used to screen for antenatal or postnatal depression in community and clinical settings (see Additional file 3) [27]. This instrument assesses feelings of guilt, sleep disturbance, anhedonia (i.e., the inability to derive pleasure from activities considered to be pleasurable) and thoughts of self-harm that have been present for the past 7 days. Each question is scored on a 4-point scale ranging from 0 to 3 points. Overall scores are generated by the sum of these responses. Scores between 10 and 12 points suggest increased risk for depression, and scores of 13 points or more suggest that the diagnostic criteria for major depression have been met [28]. In these cases, patients should undergo formal diagnostic assessment [16]. Scores of 1 point or more on question 10 of the EPDS (regardless of the overall score), also require further assessment as this question deals with self-harm.

Although NICE guidelines recommend using a two-staged approach where the EPDS is administered only if there is an affirmative answer to at least one Whooley question, all the participants in this study completed the EPDS regardless of their answers to the Whooley questions.

#### Scrolling layout

**Fig. 2:**
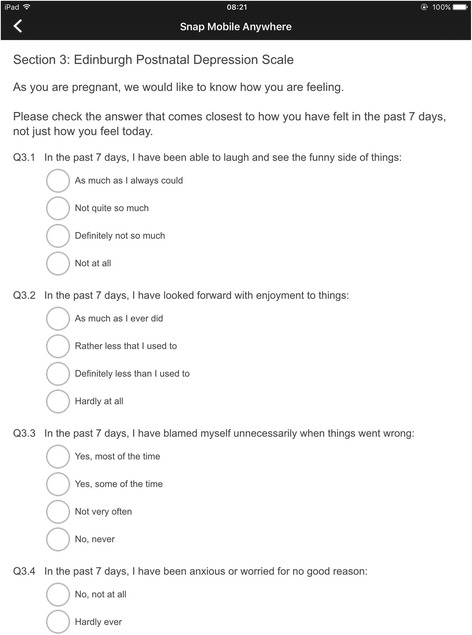
Scrolling layout - Screenshot

In this type of survey layout, each survey was presented on a single screen (see Fig. 2). Therefore, participants had to scroll vertically in order to answer all the questions. Participants were allowed to navigate vertically and between surveys, and to modify their answers to any questions before submitting their answers. After pressing the submit button, they were asked to return the tablet computer to the research midwife or CSO who was then presented with a summary of the participant’s results. This information was used to complete a summary letter, which was given to a midwife or consultant in time for the consultation. Validation rules were applied with the app, which prevented participants from leaving any questions unanswered.

#### Paging layout

**Fig. 3:**
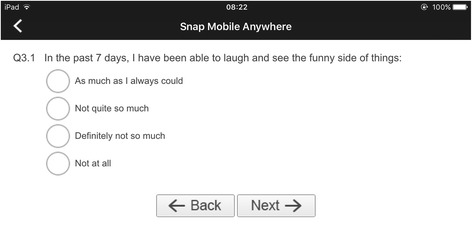
Paging layout - Screenshot

The study procedures for this type of survey layout were identical as those for the Scrolling Layout. The difference was that only one question was displayed on the screen at any given time (see Fig. 3). Participants were allowed to navigate between questions and to modify their answers before pressing the submit button.

### Study procedures

#### Randomisation

We used block randomisation (with blocks of 4) to allocate participants to one of the two manipulations of the survey layout: paging or scrolling. For each participating site, we generated an independent sequence using Stata V.13.0 [29]. These sequences were printed and placed inside opaque, numbered envelopes that were then sealed and distributed. Research midwives and CSOs were not involved in generating random sequences. Envelopes were used sequentially and opened once a participant had completed the informed consent form. The contents of the envelope informed the research midwife or CSO regarding which version of the surveys they should activate; at this point, they handed over the tablet computer to the participant. If a participant was called in before completing the surveys, her record on the tablet was cancelled and the content of that randomisation envelope discarded.

### Statistical analyses

We compared the two survey layouts on participants’ responses to the Whooley questions and the EPDS separately. To this end, we compared the proportion of participants answering *Yes* to at least one Whooley question between the *Scrolling* and *Paging* groups. We also compared these groups in relation to the median EPDS scores, the proportion of participants scoring at each interval of the EPDS (i.e., 0 to 9 points, 10 to 12 points, and 13 points or more), and the proportion of participants scoring 1 point or more on question 10 of the EPDS.

Moreover, we presented the overall median completion time (seconds) across all participants and evaluated differences on this outcome between the *Scrolling* and *Paging* groups. We also compared the proportion of participants requesting technical assistance between these groups. In addition, we measured smartphone and tablet ownership across all participants.

We used a chi-squared test to compare proportions of participants, and the Wilcoxon rank-sum test as the EPDS scores and the completion times were not normally distributed.

We conducted all our analyses using R version 3.3.1 [30].

## Results

Of 597 pregnant women who consented to participate, 543 completed the study procedures (see Fig. 1). However, we removed 13 records as they were submitted more than 24 h after completion. Therefore, we included 530 records in our analysis: 273 from the *Scrolling* group and 257 from the *Paging* group. Participants were recruited from 14 localities across England (Blackburn, Bolton, Burton, Bury, East Midlands, London, Huntingdon, North East, Oldham, Rochdale, Shrewsbury, Stoke-on-Trent, Telford, and Wigan). Table 1 provides a summary of the socio-demographic characteristics of our sample.

**Table 1:** Summary of participants’ socio-demographic characteristics

		Scrolling n(% *N* = 273)	Paging n(% *N* = 257)
Recruitment Setting	General Practice	59(21.61%)	56(21.79%)
	Community	14(5.13%)	12(4.67%)
	Hospital	200(73.26%)	189(73.54%)
Age group	18 – 22 years	15(5.49%)	11(4.28%)
	23 – 27 years	51(18.68%)	34(13.23%)
	28 – 32 years	90(32.97%)	108(42.02%)
	33 – 37 years	83(30.40%)	81(31.52%)
	38+ years	34(12.45%)	23(8.95%)
Ethnic Background	White	173(63.37%)	182(70.82%)
	Mixed	9(3.30%)	7(2.72%)
	Asian	64(23.44%)	45(17.51%)
	Black	14(5.13%)	14(5.45%)
	Other ethnic group	12(4.40%)	9(3.50%)
	Not disclosed	1(0.37%)	0(0.00%)
Relationship status	Single	17(6.23%)	13(5.06%)
	Married/Civil Partnership	180(65.93%)	168(65.37%)
	Cohabiting	71(26.01%)	67(26.07%)
	Divorced/Widowed/Separated	2(0.73%)	3(1.17%)
	Not disclosed	3(1.10%)	6(2.33%)
Employment status	Full-time employment	135(49.45%)	141(54.86%)
	Part-time employment	52(19.05%)	49(19.07%)
	Self-employed	25(9.16%)	19(7.39%)
	Seeking employment	6(2.20%)	13(5.06%)
	Unemployed	49(17.95%)	34(13.23)
	Disability	6(2.20%)	1(0.39)
Highest educational achievement	Postgraduate degree (MSc, PhD)	56(20.51%)	52(20.23%)
	University degree	107(39.19%)	94(36.58%)
	College degree or below	95(34.80%)	97(37.74%)
	None	8(2.93%)	5(1.95%)
	Other qualification	7(2.56%)	9(3.50%)

### Participants’ responses to recommended depression screening scales

#### Whooley questions

Overall, 122 (23.02%) participants answered *Yes* to at least one Whooley question and 408 (76.98%) participants answered *No* to both questions (Table 2).

**Table 2:** Number of participants according to their answers to the Whooley questions and by survey layout (i.e., Scrolling vs. Paging)

	*Yes* to one or both questions n(row %)	*No* to both questions n(row %)
Scrolling *N* = 273	62(22.71%)	211(77.29%)
Paging *N* = 257	60(23.35%)	197(76.65%)
Total *N* = 530	122(23.02%)	408(76.98%)

We performed a chi-squared test of independence to examine the relation between participants’ answers to the Whooley questions (i.e., the number of participants answering *Yes* to one or both Whooley questions and those answering *No* to both questions) and survey layout (i.e., *Scrolling* and *Paging*) (Table 2). The relation between these variables was non-significant, *χ* ^2^ (1, *N* = 530) = 0.0049718, *p* = 0.944.

#### Edinburgh postnatal depression scale

Table 3 shows the mean and median EPDS scores for all participants, as well as by survey layout allocation. We compared the median EPDS scores between both survey layouts using a Wilcoxon rank-sum test and found no difference between them: W = 35755, *p* = 0.700.

**Table 3:** Overall mean and median EPDS scores for all participants and by survey layout

	Mean EPDS score (SD)	Median EPDS score
Scrolling N = 273	4.72(4.82)	3
Paging N = 257	4.43(3.80)	3
Overall N = 530	4.58(4.35)	3

Table 4 shows the overall number of participants at each EPDS interval, as well as by survey layout allocation. We performed a chi-squared test of independence to examine the relation between EPDS scoring intervals and survey layout (Table 4). The relation between these variables was significant, *χ* ^2^ (2, *N* = 530) = 9.6779, *p* = 0.008.

**Table 4:** Overall number of participants at each EPDS interval and by survey layout

	Overall scores 0 – 9 points n(row %)	Overall scores 10 – 12 points n(row %)	Overall scores 13+ points n(row %)
Scrolling N = 273	236(86.45%)	14(5.13%)	23(8.42%)
Paging N = 257	225(87.55%)	24(9.34%)	8(3.11%)
Total N = 530	461(86.98%)	38(7.17%)	31(5.85%)

Table 5 shows the overall number of participants by their score on question 10 of the EPDS, as well as by survey layout allocation. We performed a chi-squared test of independence to examine the relation between participants’ scores on question 10 of the EPDS and survey layout (i.e., *Scrolling* and *Paging*) (Table 5). The relation between these variables was non-significant *χ* ^2^ (1, *N* = 530) = 0.010247, *p* = 0.919.

**Table 5:** Number of participants by score on question 10 of the EPDS and by survey layout

	0 points on question 10 n(row %)	1 point or more on question 10 n(row %)
Scrolling *N* = 273	268(98.17%)	5(1.83%)
Paging *N* = 257	251(97.67%)	6(2.33%)

### Completion time and requests for technical assistance

For our calculations concerning completion time, we excluded participants who requested technical help from the local research team during survey completion. Therefore, we included data from 495 participants: 254 from the *Scrolling* group and 241 participants from the *Paging* group.

Overall, 75% of participants completed the study procedures in 7 min 16 s or less, and half of them in 5 min 9 s or less. These completion times included the baseline demographic data survey, and are thus an overestimation of the real duration of the screening process (i.e., Whooley questions and EPDS). To give a sense of the overestimation, we trialled the Whooley questions and the EPDS with colleagues, which yielded estimates of 2 min 28 s. Using a Wilcoxon rank-sum test, we observed that participants in the *Scrolling* group were faster at completing the surveys (Mdn = 285.5 s) than participants in the *Paging* group (Mdn = 333 s): W = 27014, *p* = 0.024.

Overall, 35 participants made requests for technical assistance. We performed a chi-squared test of independence to explore the relation between the number of participants asking for technical assistance and survey layout (i.e., *Scrolling* and *Paging*) (Table 6). The relation between these variables was non-significant *χ* ^2^ (1, *N* = 530) = 0.027251, *p* = 0.869.

**Table 6:** Number of participants by requests for technical assistance and by experimental group

	No requests n(row %)	Requests for technical assistance n(row %)
Scrolling *N* = 273	254(93.04%)	19(6.96%)
Paging *N* = 257	241(93.77%)	16(6.23%)
Total *N* = 530	495(93.40%)	35(6.60%)

No participant made more than one request for technical assistance. In addition, Table 7 provides a summary of the types of requests made.

**Table 7:** Types of requests for technical assistance by experimental group

Type of request	Scrolling – number of requests (% of 19 requests)	Paging – number of requests (% of 16 requests)	Row total (% of 35 requests across both groups)
Clarification of question	14 (73.68%)	11 (68.75%)	25 (71.43%)
Interface issues	1 (5.26%)	1 (6.25%)	2 (5.71%)
Navigation issues	0 (0%)	3 (18.75%)	3 (8.57%)
Not recorded	4 (21.05%)	1 (6.25%)	5 (14.29%)

### Smartphone and tablet computer ownership

From the 530 participants included in the analysis, 518 (97.74%) reported owning a smartphone and 435 (82.10%) reported owning a tablet computer.

## Discussion

### Participants’ answers to the Whooley questions and the EPDS

In this study we assessed the feasibility of using tablet computers in the waiting area of antenatal clinics for implementing NICE recommendations for the recognition of antenatal depression. Concerning participants’ responses to the depression screening scales, we observed that approximately 23% of participants answered *Yes* to at least one Whooley question. In addition, approximately 13% of participants scored 10 points or more on the EPDS. These are screening scales and, as such, do not provide a diagnosis of depression. However, these figures are within the expected prevalence rates of antenatal depression. Therefore, our findings suggest that depression screening using tablet computers may not result in a disproportionate number of patients screening positive.

Moreover, we compared two survey layouts (i.e., Scrolling vs Paging) on participants’ responses to the Whooley questions and the EPDS. We observed no relation between survey layout (i.e., *Scrolling* and *Paging*) and participants’ answers to the Whooley questions, participants’ median EPDS scores, and participants’ answers to question 10 of the EPDS (which deals with thoughts of self-harm). However, we found a significant relationship between survey layout and EPDS scoring thresholds.

The current study design does not allow us to determine if these findings are the result of a survey layout effect or a difference in the proportion of participants with clinical depression. Particularly, since we did not conduct diagnostic procedures. However, either possibility should be explored further to avoid any risks of missing mothers-to-be who might have met diagnostic criteria for major depression due to changes in the layout of antenatal depression screening scales. Alternatively, these findings could represent a baseline imbalance in another factor not captured by our baseline survey, or an increased number of false positives.

### Completion time and participants’ request for technical assistance

In this study, we chose completion time and participants’ requests for technical assistance as indicators of the potential added burden of mobile-based depression screening on the workload and schedules of clinical staff.

With regard to completion time, most participants (75%) completed the surveys under 7 min 16 s. This measure however, included the socio-demographic survey (which is not a component of depression screening) and is an overestimation of the real duration of the screening process. Therefore, we considered the observed completion times in relation to our internal trials of the Whooley questions and the EPDS alone (i.e., 2 min 28 s). This suggests that the implementation of this type of depression screening might cause minimal disruption to a clinics’ schedules. Furthermore, we found that participants in the *Scrolling* group were significantly faster than participants in the *Paging* group. This difference of 47.5 s may not be meaningful given the speed of completion.

Approximately 7% of all participants requested technical assistance during survey completion, which amounted to 35 unique requests (as no participant made more than one request). We found that the choice of survey layout did not affect the proportion of participants asking for help in each group. About 71% of these requests (25 of 35) were to clarify the meaning of questions. The remaining 10 requests were related to difficulties navigating through the surveys (due to the implementation of validation rules that prevented participants from progressing through the surveys leaving questions unanswered) and interacting with the user interface (namely, interacting with the response options). These findings suggest that this population is already familiar with mobile technology, also implied by the level of smartphone and tablet ownership in our sample (approximately 98% and 82%, respectively), and that there is no need for clinical members of staff to oversee the survey completion process. Instead, these requests could be dealt with by non-clinical support staff (as long as patient have the opportunity to discuss their screening results with a clinician during their appointment).

### Deployment challenges identified during the feasibility study

A perceived concern that has been reported in the literature is the location and privacy of screening [5, 22]. This concern was also voiced by many clinicians at the various recruitment sites prior initiating the study. We chose recruitment and completion rates as indicators of whether participants felt comfortable undergoing depression screening in the waiting area of antenatal clinics. We found that most eligible mothers-to-be who were approached (approximately 90%) agreed to take part in the study, and approximately 91% of those who took part completed the study procedures. In those sites where the research midwives or CSOs recorded the reasons given by eligible mothers-to-be for refusing to take part in the study, we found that they expressed a lack of interest in the study and not wanting to miss being called to their appointment. Moreover, the only reason that some participants could not complete the study procedures (i.e., breakoff) was that they were called in to their appointment. These findings suggest that mobile technology may offer sufficient privacy and anonymity to patients, so that they are able to complete depression screening scales in a clinic’s waiting area before their antenatal appointment.

We initially intended to use breakoff rates (and the corresponding reasons) as an indicator of participants’ perceived intrusiveness of the screening process. Since participants suspended their participation only if they were called in to their appointment, this outcome became an indicator of the compatibility between clinical workflows and research procedures. Overall, 133 women were called in to their appointment at different stages of the study: 44 (33%) before being approached by a research midwife or CSO, 35 (26%) before they were able to complete the consent process, and 54 (41%) before they were able to complete the study procedures.

The main difficulty during the initial stage was researchers’ lack of time or capacity. Most women called in before being approached about participation (37 of 44 or approximately 84%) were in primary care facilities with short waiting times between patient *check in* and appointments. This presented a challenge for research midwives and CSOs working in these facilities, as they struggled to recruit enough participants into the study. The remaining women (7 of 44 or approximately 16%) were in secondary care sites with a larger volume of patients and longer waiting times. In these facilities however, research midwives and CSOs did not have the capacity to approach all the eligible mothers-to-be who were identified.

Although most women who were called in during the consent process or the study procedures were in secondary care facilities (73 of 89 – 82%), the main difficulty during these stages was the incompatibility between the administrative research procedures and clinical workflows. We have already discussed how the socio-demographic survey (which clinical care teams would not need to administer as they should have access to this information) inflated the real duration of the screening process. However, we also need to consider the additional time required to conduct non-routine, non-clinical procedures and their impact on clinical workflows: providing information about the study, discussing a participant information leaflet, answering any questions that potential participants might have, and completing an informed consent form.

These findings highlight the tension between the need for sound evidence about the impact of technology on antenatal depression screening, and the challenges associated with introducing these technologies in clinical settings. They also highlight that the planning of antenatal depression screening through mobile devices must be sensitive to local clinical workflows, and to the role of the staff members (both clinical and non-clinical) who will be responsible for their implementation.

The introduction of mobile technology for antenatal depression screening in clinical settings also needs to take into consideration issues of connectivity. Wi-Fi access varied between the participating clinics. This was a key determinant for our choice of app, as we needed support for offline data collection. We suspect that this might also represent a requirement for clinical teams wishing to implement this type of depression screening. In this context however, there is an added issue concerning the timely transmission of patient results to clinicians. In our study, this was done through a paper form that research midwives and CSOs filled in as soon as participants completed the study. This would introduce an extra administrative step to the routine activities of a clinic, not taking full advantage of the internet capabilities of mobile devices to facilitate data management. Timely transmission of patient results could be a problem without interoperability between the mobile app and clinical systems.

Lastly, the introduction of mobile technology in clinical settings also needs to take into account the management of technology. Security is a key concern in this context, particularly in relation to lost devices or theft. In addition to the economic implications, this could also lead to a breach of confidentiality, if patient data are held on the devices. Therefore, clinical teams should ensure compliance with their organisation’s information governance policies without affecting patient experience. For example, passwords are considered ideal for protecting sensitive data. However, passwords could also disrupt clinical workflows if patients using password-protected devices time out and have to request help from staff members. Clinical teams also need to ensure that data are stored and transmitted using approved data encryption standards. Moreover, those responsible for the devices must be aware of any software update containing security patches.

### Limitations

We recruited adult participants who felt comfortable reading and writing in English. However, important risk factors for antenatal depression include young age and individuals from minority groups. We relied on self-disclosure when assessing the eligibility of potential participants. We did not perform diagnostic interviews to assess a diagnosis of depressive disorder, and this limits our ability to determine if there was a true survey layout effect on participants’ responses to the depression screening scales. Our measures of completion time included the socio-demographic baseline survey, which is not a component of antenatal depression screening. Therefore, the inclusion of this survey inflated the true completion times. We considered indirect measures of the impact of mobile technology on the activities of antenatal clinics. In some sites, the study procedures were administered by research staff with no links to the clinical team, or by research midwives with dual responsibilities: clinical and research. In addition, the administrative research requirements were unnatural to the reality of antenatal clinics and, in some cases, caused disruption.

## Conclusions

It is feasible to use tablet computers for the administration of validated screening depression scales in the waiting area of antenatal clinics. Survey layout did not influence the proportion of mothers-to-be that screen positive using the Whooley questions or their EPDS scores in this study. However, we observed a significant relationship between survey layout and EPDS scoring intervals. This association should be explored further to determine if it corresponds to a survey layout effect, in order to ensure that clinically depressed mothers-to-be are not missed due to non-clinical effects. The use of mobile technology for antenatal depression screening does not seem to disrupt the routine activities of antenatal clinics. Nonetheless, it is crucial that practitioners and commissioners consider the successful integration of this technology into existing clinical workflows, the timing of appointment, as well as connectivity and security issues. These findings are relevant in the context of the design and implementation factors that could affect the quality of the responses given to validated depression screening scales.

## Additional files

Additional file 1: Socio-demographic survey. Description of data: eleven-question survey used to gather socio-demographic information from participants. (DOCX 66 kb)

Additional file 2: Whooley Questions. Description of data: two-item survey used to evaluate the presence of depressive mood and anhedonia over the past month. (DOCX 46 kb)

Additional file 3: Edinburgh Postnatal Depression Scale. Description of data: a ten-item instrument used to screen for antenatal or postnatal depression in community and clinical settings. This instrument assesses feelings of guilt, sleep disturbance, anhedonia and thoughts of self-harm that have been present for the past 7 days. (DOCX 84 kb)

## Abbreviations

CSO: Clinical studies office
EPDS: Edinburgh postnatal depression scale
iOS: iPhone operating system
NHS: National health service
NICE: The National Institute for health and care excellence
PIL: Participant information leaflet
SD: Standard deviation
