# Supplementary material for: Implementation of depression screening in antenatal clinics through tablet computers: results of a feasibility study
Source: BMC Med Inform Decis Mak. 2017 May 10;17:59. doi: 10.1186/s12911-017-0459-8 (PMC5424386; doi:10.1186/s12911-017-0459-8)
Supplement: Supplementary file 1 — Socio-demographic survey. Description of data: eleven-question survey used to gather socio-demographic information from participants. (DOCX 66 kb) [file 12911_2017_459_MOESM1_ESM.docx]

# Appendix 1. Socio-demographic survey

1. What is your age group?

- 18 to 22 years
- 23 to 27 years
- 28 to 32 years
- 33 to 37 years
- 38 years or older

1. How would you describe your race/ethnicity?

- White British
- White Irish
- Other White
- White and Black Caribbean
- White and Black African
- White and Asian
- Other Mixed
- Indian
- Pakistani
- Bangladeshi
- Other Asian
- Black Caribbean
- Black African
- Other Black or Black British
- Chinese
- Other Ethnic Group
- Not stated

1. What is your marital status?

- Single
- Married/In civil partnership
- Divorced/Civil partnership that has been dissolved
- Widowed
- Separated
- Not stated

1. What is your employment status?

- Employed, full-time
- Employed, part-time
- Self-employed
- Not employed, looking for work
- Not employed, not looking for work
- Disability/Not able to work

1. What is your highest level of education?

- University or college degree
- University or college qualification below degree level
- A Levels or equivalent
- GCSE or equivalent
- None of these

1. Do you own a smartphone (e.g., iPhone, Samsung, Blackberry, HTC, Sony)?

- Yes
- No

1. Do you own a tablet computer (e.g., iPad, iPad mini, Samsung tablet)?

- Yes
- No

1. Is this your first pregnancy?

- Yes
- No

1. (If answer to previous question is *No*) How many children have you given birth to?
2. When is your current baby due?
3. Have you ever been diagnosed with depression?

- Yes
- No
