# Supplementary material for: Implementation of depression screening in antenatal clinics through tablet computers: results of a feasibility study
Source: BMC Med Inform Decis Mak. 2017 May 10;17:59. doi: 10.1186/s12911-017-0459-8 (PMC5424386; doi:10.1186/s12911-017-0459-8)
Supplement: Supplementary file 2 — Whooley Questions. Description of data: two-item survey used to evaluate the presence of depressive mood and anhedonia over the past month. (DOCX 46 kb) [file 12911_2017_459_MOESM2_ESM.docx]

# Appendix 2. Whooley questions

1. Over the past month, have you been bothered by feeling down, depressed or hopeless?

- Yes
- No

1. Over the past month, have you been bothered by having little interest or pleasure in doing things?

- Yes
- No

Source:

Whooley MA, Avins AL, Miranda J, Browner WS. Case-finding instruments for depression – two questions are as good as many. J Gen Intern Med 1997;12:439-45.
