# Supplementary material for: Implementation of depression screening in antenatal clinics through tablet computers: results of a feasibility study
Source: BMC Med Inform Decis Mak. 2017 May 10;17:59. doi: 10.1186/s12911-017-0459-8 (PMC5424386; doi:10.1186/s12911-017-0459-8)
Supplement: Supplementary file 3 — Edinburgh Postnatal Depression Scale. Description of data: a ten-item instrument used to screen for antenatal or postnatal depression in community and clinical settings. This instrument assesses feelings of guilt, sleep disturbance, anhedonia and thoughts of self-harm that have been present for the past 7 days. (DOCX 84 kb) [file 12911_2017_459_MOESM3_ESM.docx]

# Appendix 3. Edinburgh Postnatal Depression Scale

As you are pregnant or have recently had a baby, we would like to know how you are feeling. Please check the answer that comes closest to how you have felt IN THE PAST 7 DAYS, not just how you feel today

In the past 7 days:

1. I have been able to laugh and see the funny side of things

- As much as I always could
- Not quite so much now
- Definitely not so much now
- Not at all

1. I have looked forward with enjoyment to things

- As much as I ever did
- Rather less than I used to
- Definitely less than I used to
- Hardly at all

1. I have blamed myself unnecessarily when things went wrong

- Yes, most of the time
- Yes, some of the time
- Not very often
- No, never

1. I have been anxious or worried for no good reason

- No, not at all
- Hardly ever
- Yes, sometimes
- Yes, very often

1. I have felt scared or panicky for no very good reason

- Yes, quite a lot
- Yes, sometimes
- No, not much
- No, not at all

1. Things have been getting on top of me

- Yes, most of the time I haven’t been able to cope at all
- Yes, sometimes I haven’t been coping as well as usual
- No, most of the time I have coped quite well
- No, I have been coping as well as ever

1. I have been so unhappy that I have had difficulty sleeping

- Yes, most of the time
- Yes, sometimes
- Not very often
- No, not at all

1. I have felt sad or miserable

- Yes, most of the time
- Yes, quite often
- Not very often
- No, not at all

1. I have been so unhappy that I have been crying

- Yes, most of the time
- Yes, quite often
- Only occasionally
- No, never

1. The thought of harming myself has occurred to me

- Yes, quite often
- Sometimes
- Hardly ever
- Never

Sources:

Cox JL, Holden JM, Sagovsky R. Detection of postnatal depression: Development of the 10-item Edinburgh Postnatal Depression Scale. *British Journal of Psychiatry 1987*; 150:782-786.

Wisner KL, Parry BL, Piontek CM. Postpartum Depression. N Engl J Med 2002; 347(3): 194-199.
